# Indian adolescents’ perceptions of the home food environment

**DOI:** 10.1186/s12889-018-5083-8

**Published:** 2018-01-22

**Authors:** Neha Rathi, Lynn Riddell, Anthony Worsley

**Affiliations:** Institute for Physical Activity and Nutrition, Deakin University, Geelong, VIC Australia

**Keywords:** Home food environment, Adolescents, India

## Abstract

**Background:**

The home food environment has the potential to influence the eating behaviour of adolescents. This investigation aimed to understand Indian adolescents’ perspectives of their home food environments.

**Methods:**

Adolescents aged 14–16 years (*n* = 1026, 65.3% girls) attending private secondary schools in Kolkata completed a paper-based questionnaire during school time which included questions about family food rules, availability and accessibility of foods at home, and domestic cooking responsibility. Boys’ and girls’ opinions and experiences were compared through cross-tabulation analyses.

**Results:**

Almost all the adolescents reported that fruits (91.6%) and vegetables (95.7%) were always available in their homes. Approximately two-fifths reported that sugar-sweetened beverages (36.2%) and salty snack foods (38.0%) were readily available. In 56.1% households, adolescents were expected to follow certain food rules during mealtimes (e.g. not talking with my mouth full). The majority of the respondents (80.4%) identified mothers as the primary meal providers, only a minority reported that fathers (5.1%) were responsible for preparation of family meals.

**Conclusion:**

This understanding of the family-environmental determinants of adolescent dietary habits provides useful directions for nutrition promotion interventions. Health and educational professionals associated with adolescents could communicate about the development of healthy home food environments to provide positive health benefits for adolescents and their families.

## Background

The rapid emergence of adolescent obesity in India over the last decade [1, 2] has led to increasing concern about the diets of adolescents [3]. India adolescents’ diets appear to be characterised by over-consumption of energy-dense, nutrient-poor foods and sugar-sweetened beverages as well as low intakes of fruits and vegetables [4, 5]. These poor dietary intakes can have a significant impact on both immediate and long-term health of adolescents [6].

A healthy diet can substantially reduce the risk of obesity and obesity-related morbidities, such as diabetes, hypertension, a range of carcinomas and coronary disease [3]. For adolescents, a healthy diet also contributes to physiological growth and development [7]. Since food habits adopted during adolescents are likely to be maintained during adulthood [8], it is important to identify the correlates of adolescent dietary behaviours to assist in development of successful interventions to address adolescent obesity [9].

Several factors appear to influence the development of adolescent dietary practices. These include individual (e.g. taste, nutrition-related knowledge, self-efficacy) social environmental (e.g. parents, peers), physical environmental (e.g. school canteen, fast food establishments), and macrosystem influences (e.g. food advertisements) [9–13].

Empirical evidence clearly highlights the importance of parents and home environment as a key component of healthy adolescent development [14–16]. During adolescence, parents continue to function as primary food gatekeepers for their families [11, 13]. Adolescents meet about two-fifths of their daily nutritional needs from foods available at home [17]. In addition, data from developed countries like the US [18] and New Zealand [19] suggest that 30% - 42% adolescents consume one family meal daily at home. Moreover, Neumark-Sztainer and colleagues reported that nearly three-quarters of adolescents (*n* = 252) enjoyed eating meals at home in the company of their family members [18] highlighting, the significance of home environment in adolescent nutrition.

Food-related parenting practices [20], parental role modelling [21], provision of food within the home environment [22], and eating meals as a family [23] have been recognised in Western countries as powerful predictors of food consumption among adolescents. For example, the availability of unhealthy savoury snacks at home may be associated with increased intake among adolescents [22]. Similarly, the opportunity for adolescents to eat meals with their families has been associated with healthy eating attitudes and behaviours [23, 24]. Authoritarian feeding styles (in which parents compel adolescents to consume certain foods or complete meals) have been associated with lower intake of nutritious foods [20] but high consumption of energy-dense, nutrient-poor snacks [25] and greater weight gain [26] in young people.

While the evidence regarding adolescents’ home food environments is expanding, the majority of research to date has been conducted in economically developed countries. There is currently a paucity of literature examining parental influence and home food environments in the Indian context. To our knowledge, only one qualitative study has explored the influences on Indian adolescents’ dietary behaviours [27]. Parental and peer influences, home and school food environments, as well as mass media emerged as key perceived influences on adolescent dietary behaviours [27]. Therefore, the aim of this study is to better understand Indian adolescents’ home food environments using quantitative techniques within a broad sample of adolescents. Evidence suggests that there are gender-based differences in young people’s attitudes towards health and food-related issues, females tending to exhibit healthier attitudes and dietary habits than males [28, 29]. Therefore, based on this evidence it is important to evaluate gender differences on the household environment variables.

## Methods

### Study design and sampling

The present investigation draws on cross-sectional data from a sample of 1026 Indian adolescents who participated in the “Dietary and Lifestyle” (DAL) survey. DAL survey details have been reported previously [30–32], and are also outlined below. The survey protocol received ethical approval from Deakin University’s Health Ethics Advisory Group (HEAG-H 187_2014).

The sampling frame for this investigation consisted of year nine students (aged 14–16 years) studying in English-speaking, private secondary schools in Kolkata, India. Through convenience sampling, private secondary schools (*n* = 9; two co-educational schools, two single-sex boys’ schools, and five single-sex girls’ schools) were selected because obesity is more prevalent among private school students when compared to government school students [33]. Moreover, private schools are responsible for delivering secondary education to 40% adolescents in urban India [34].

### Survey instrument

The students were asked to complete the Dietary and Lifestyle Questionnaire (DALQ) during school hours. This 15-page, self-reported instrument included a number of measures (*n* = 17) of the home food environment which were developed after a review of the literature [18, 22, 35, 36] and 52 face-to-face interviews with adolescents, parents, teachers and school principals [27]. Five statements were used to elicit adolescents’ perceptions of family food rules. Two questions asked about food accessibility at home and seven items enquired about the availability of food at home. Three items relating to domestic cooking responsibility were also listed in the DALQ. All these items used four-point response scales ranging from “never” (1), “sometimes” (2), “usually” (3), and “always” (4). In addition, three items relating to the students’ age, gender, and religion were included. Categorical responses were used for these demographic measures. For example, the question about the ages of the respondents had three response categories: “14 years” (1), “15 years” (2), and “16 years” (3). Prior to conducting the full-scale survey, the survey measures were pre-tested among a convenience sample of 47 adolescents to ensure their clarity. This was followed by the reliability study where 37 adolescents completed the survey twice with a test-retest interval of four weeks. The correlation coefficient (r) values for the test-retest reliability of the 17 home food environment measures discussed in the present context are indicated in Table 1.

**Table 1:** Correlation coefficient (r) values for test-retest reliability of home food environment measures^a^

Measure	r (Pearson’s correlation)
Family food rules	
I’m allowed to buy whatever I want from fast food joints	0.85
I can eat whatever I like at home	0.87
During meal times, I’m allowed to put the TV on	0.79
I’m expected to eat all the foods served even if I don’t like them	0.89
At mealtimes I have to follow certain rules	0.91
Accessibility to food at home	
Vegetables are served at dinner	0.84
There is plenty of food at home	0.58
Availability of food at home	
Potato chips or other salty snack foods	0.64
Soft drink (e.g. Coke)	0.82
Chocolate or other lollies (sweets)	0.88
Cakes/pastries/donuts/biscuits	0.71
Fruits	0.92
Vegetables	0.72
Fruit juice	0.76
Domestic cooking responsibility	
The domestic help makes all the food at home	0.56
My mother makes all the food at home	0.66
My father makes all the food at home	0.35

^a^All the correlations except for the last item i.e. “My father makes all the food at home” were statistically significant at *p* <  0.01

### Procedure

A detailed explanation of the survey methods was presented to the school authorities in person. During morning assemblies, the school authorities made announcements to invite all year nine students (*n* = 1095) to complete the survey. A recruitment pack was sent to students’ homes for parental approval. In total, 1079 parents provided written consent for their adolescent’s participation. Subsequently, 1026 students completed the survey in the presence of teachers and the researcher on school premises between December 2015 and April 2016 (i.e. response rate 93.6% of adolescents invited to take part). This sample size assured adequate power for the study (i.e. a power of 97%), an effect size of 0.3 (Cohen’s *d*), and a significance level of .01. The respondents did not receive any gifts or incentives for their participation.

### Data analysis

After examining for completeness and consistency, the questionnaire data was entered into the Statistical package for Social Sciences (SPSS version 22.0, IBM Corp., Armonk, NY, 2013) which was used for data analyses. Descriptive statistics were calculated to summarise the variables. After inspection of the data the four point-response scales were merged to form two categories ‘never/sometimes’ and ‘usually/always’ for cross-tabulations. Cross-tabulations were used to evaluate gender differences on the environment variables. Statistical significance was set at *p*-value < .01.

## Results

### Demographic characteristics

Nearly, two-thirds of the adolescents (65.3%) were girls, the majority of the sample was aged between 14 and 15 years (86.1%), with the remainder (13.9%) reporting their age as 16 years. Hinduism (70.7%) was identified as the most common religion.

### Family food rules

Approximately three fifth of the participants (58.8%) stated that they were allowed to eat foods of their choice at home (Table 2). In 56 % of homes, adolescents were required to adhere to certain food rules during the course of their meals (e.g. not talking with my mouth full). However, nearly two thirds of the adolescents (64.6%) reported that they did not have a family food rule prohibiting television viewing during meal times. On the whole, the cross-tabulation analyses indicate that the prevalence of family meal time rules did not significantly vary between boys’ and girls’ homes. Nonetheless, a significantly higher proportion of boys (59.3%) than girls (48.7%) reported that they were expected to eat all the foods served even if they did not like them (*p* < .01).

**Table 2:** Adolescents’ perceptions of the home food environment (% Always^a^, *n* = 1026)

	Boys % (*n*)	Girls % (*n*)	Total % (*n*)	χ^2^	df	*p*-value
Family food rules						
I’m allowed to buy whatever I want from fast food joints	31.7 (113)	23.7 (159)	26.5 (272)	7.656	1	< 0.01
I can eat whatever I like at home	62.6 (223)	56.7 (380)	58.8 (603)	3.367	1	0.06
During meal times, I’m allowed to put the TV on	67.7 (241)	63.0 (422)	64.6 (663)	2.257	1	0.13
I’m expected to eat all the foods served even if I don’t like them	59.3 (211)	48.7 (326)	52.3 (537)	10.497	1	< 0.01
At mealtimes I have to follow certain rules	57.9 (206)	55.2 (370)	56.1 (576)	0.659	1	0.42
Accessibility to food at home						
Vegetables are served at dinner	82.0 (292)	73.9 (495)	76.7 (787)	8.625	1	< 0.01
There is plenty of food at home	86.2 (307)	81.8 (548)	83.3 (855)	3.307	1	0.06
Availability of food at home						
Potato chips or other salty snack foods	39.0 (139)	37.5 (251)	38.0 (390)	0.247	1	0.61
Soft drink (e.g. Coke)	43.0 (153)	32.5 (218)	36.2 (371)	10.977	1	< 0.01
Chocolate or other lollies (sweets)	47.2 (168)	46.6 (312)	46.8 (480)	0.036	1	0.84
Cakes/pastries/donuts/biscuits	43.8 (156)	50.6 (339)	48.2 (495)	4.276	1	0.03
Fruits	93.3 (332)	90.7 (608)	91.6 (940)	1.910	1	0.16
Vegetables	94.7 (337)	96.3 (645)	95.7 (982)	1.460	1	0.22
Fruit juice	60.1 (214)	53.0 (355)	55.5 (569)	4.781	1	0.02
Domestic cooking responsibility						
The domestic help makes all the food at home	21.9 (78)	16.6 (111)	18.4 (189)	4.416	1	0.04
My mother makes all the food at home	80.1 (285)	80.6 (540)	80.4 (825)	0.043	1	0.83
My father makes all the food at home	6.5 (23)	4.3 (29)	5.1 (52)	2.197	1	0.14

^a^Always = usually (3) + always (4) = 4

### Accessibility of food at home

In the majority of the households (83.3%), an adequate amount of food was available all the time (Table 2). Around three fourth of the participants (76.7%) revealed that vegetables were regularly served at dinner. Both the male and female respondents had similar views of food accessibility.

### Availability of food at home

Fruits (91.6%) and vegetables (95.7%) were the most frequently available foods in students’ homes (Table 2). Salty snack foods like potato chips (38.0%) and soft drink (36.2%) were the least regularly available food items in homes. The cross-tabulation analyses indicated that the availability of various food items did not significantly vary in the homes of boys compared to the homes of girls. Nonetheless, more boys (43.0%) than girls (32.5%) indicated that sugar-sweetened beverages were available at home (*p* < .01).

### Domestic cooking responsibility

The individuals most likely to be responsible for household meal preparation were the adolescents’ mothers (80.4%) whereas fathers (5.1%) were least likely to be responsible for domestic cooking (Table 2). However, around one fifth of the respondents (18.4%) reported that the domestic help was in-charge of household cooking. No statistically significant differences in the perceptions of boys and girls were found.

## Discussion

The present investigation reports novel findings about the adolescents’ home food environment in Kolkata, India. There were four key findings: the easy accessibility of food at all times; the availability of both fresh fruits and vegetables and of nutrient-poor snacks and beverages in adolescents’ homes; the enforcement of food rules during meal times; and the finding that generally adolescents’ mothers acted as primary household food preparers. Since adolescents obtain 60% of their daily energy from meals sourced at home [17], these findings further reinforce the important influence of the home food environment on adolescents’ food habits. In contrast to many studies conducted among adults [37, 38], the present findings represent teenagers’ views of the adolescents’ home food environment. Moreover, the perceptions of teenagers are expected to be more significant and valid than those of adults because the dietary intake of teenagers is more highly associated with their own perceptions than those of their parents [39].

The availability and accessibility of foods at home is an important predictor of adolescent diet quality [9, 27, 40–42]. Nearly all the respondents claimed that fruits and vegetables were always available at home. Comparable findings were reported in Project EAT (Eating Among Teens) in the US [41] and in the YEP (Youth Eating Patterns) study in Australia [42]. The availability of healthy foods in the home has been positively associated with higher diet quality among young people [9, 40, 41]. Moreover, the household availability of fruits and vegetables is negatively associated with the consumption of sugar-sweetened beverages and snacks among American adolescents [40]. In addition, vegetables were served at dinner in about three quarter of the study respondents’ homes. Similarly, MacFarlane and colleagues noted that vegetables were always served for dinner in 61% of Australian homes (*n* = 3264) [42].

Nevertheless, nearly two-fifths of the adolescents reported the availability of sugar-sweetened beverages and potato chips in their homes. These findings are similar to previous reports [19, 42]. For example, about one-third of Australian adolescents (n = 3264) reported that soft drink and salty snacks were usually available in their homes [42]. There is compelling evidence that the presence of sugar-sweetened beverages and energy-dense, nutrient-poor snacks in the home has a negative impact on the diet quality of children and adolescents [9, 22, 43]. For example, a study of American school children (aged 8–13 years) found that students who reported that carbonated beverages were available in their households were more likely to report consuming these beverages five or more times per week [44]. One implication of this finding is that Indian parents (and parents elsewhere) might implement a covert restriction strategy i.e. not having soft drink and unhealthy snacks available or accessible at home. This might be an effective way to restrict the intake of such unhealthy foods [45].

In addition to home food availability, family food rules may also play an important role in determining young people’s food behaviours [9, 18, 42]. Approximately, three-fifths of the students in the present study were expected to follow specific food rules at meals. In contrast, only one-third of Australian adolescents (*n* = 3264) [42] and 48% of American adolescents (*n* = 233–237) [18] were expected to do the same (Both these studies used the same item (“not talking with my mouth full”) as the present study). Again, in comparison to the present findings (52%), only 35% of American adolescents were expected to consume all the foods served even if they did not like them [18]. Compulsion to consume certain foods, predominantly healthy foods, or finish meals has been associated with poor diet quality in Western cultures (e.g. the US, the UK) [20, 25] i.e. reduced intake of healthy foods (e.g. soup) [20] but greater intake of nutrient-poor snacks [25]. Perhaps, the variation in responses might be explained by the different parenting styles followed in different cultures. It is quite possible that Indian parents practiced more stringent mealtime food rules than British and American parents. Interestingly, findings from a recent qualitative investigation [27] suggest that Indian parents predominantly practice authoritative and authoritarian parenting styles similar to parents of Western cultures.

Traditionally, Indian women were entrusted with duties of household cooking [46, 47] and this is quite evident in the present investigation as the majority of the respondents’ mothers were responsible for cooking family meals. This finding is in line with previous research in which Australian [22] and Peruvian adolescents [11] made similar observations of their mothers. Mothers have been recognised as primary dietary gatekeepers within the home environment who influence the food habits of the family members and subsequently their health status [48].

However, with the advent of globalisation, the age-old extended family structure in India has undergone a major transformation [49]. The rapid emergence of nuclear families [50] has led to increased female paid employment outside the home [51]. This might partly explain the role of domestic help in household cooking in about one-fifth of the adolescents’ homes. Probably, the mothers may have used their dietary knowledge to guide their domestic help in meal preparation. However, this needs to be confirmed in further investigations. Interestingly, mothers’ dietary knowledge and practices play a key role in determining their feeding practices [22]. Nevertheless, the present findings do not show how many of the 80% of mothers were employed outside home and how they managed to find time for cooking. In general, lack of time arising out of busy work schedules has been criticised for preventing cooking at home [52–54]. Moreover, whether the domestic help were equipped with appropriate declarative and procedural nutritional knowledge also remains unknown. Therefore, more research is needed to explore these issues which are vital to the evaluation of the home food environment.

### Strengths and limitations

The strengths of this investigation include its large sample size, high response rate (93.6%) and novel findings. To the best of our knowledge, this is the first and the largest survey on home food environments of adolescents in the Indian context. The study has at least three limitations. First, its cross-sectional design does not allow for the assessment of causal relationships between the variables. Future use of longitudinal or experimental designs would allow for examination of likely causal influences on adolescents’ dietary practices. Second, convenience sampling was used and hence the sample may not represent the broader Indian adolescent community. Because of logistic reasons, random sampling could not be implemented in the current investigation. Third, the focus on year nine adolescents attending private English-speaking schools in Kolkata, especially given the size and diversity of the Indian population could have limited the generalisability of the findings. Future studies should include adolescents of different age groups from public and private schools in both rural and urban areas in different states of India.

### Implications for research and practice

Future research on home food environments should focus on assessing the views of Indian parents about family food rules, family mealtime episodes, and home food availability. The examination of different measures including adolescents’ BMI, family income, parental educational status, parents’ employment status (e.g. working from home), the household availability of both healthy and unhealthy snacks and their impact on the home food environment should be considered in future research. The assessment of these variables is important for the development of nutrition promotion initiatives in home food environments. Parents may have different views of the family-environmental determinants of adolescent nutrition compared to their adolescents [39]. The views of both adolescents and parents are essential for the effective implementation of family-oriented nutrition promotion strategies. Since, mothers act as the main meal providers for their adolescents and their families, it is important to investigate their capabilities with regards to cooking and nutritional confidence, and their food acquisition and food transformation practices. In addition, there is a need to replicate this study in a matriarchal society, such as that in Kerala, India [55], where mothers may not function as the primary dietary gatekeepers. Other possible areas for exploration include the assessment of the frequency of family meals consumed across breakfast, lunch and dinner; role modelling by parents; and the dietary knowledge and practices of mothers and fathers in matriarchal societies. Health and educational professionals associated with adolescents and their families should be aware of the barriers and facilitators of family-environmental measures on dietary habits so that they can educate their clients accordingly. There is also scope for providing public education on the development of healthy home food environments to provide positive health benefits for adolescents and their families.

## Conclusion

The present investigation provides novel insights into the home food environments of Indian adolescents. Notable findings include the easy availability and accessibility of both healthy and unhealthy foods at home, and the continuing role of mothers as the primary food gatekeepers for adolescents. These findings provide support for the development of prospective nutrition promotion strategies targeting the eating behaviours of 243 million Indian adolescents and their families.

## Abbreviations

DAL: Dietary and lifestyle
DALQ: Dietary and lifestyle questionnaire
HEAG: Health Ethics Advisory Group
